# Survey of bluetongue virus infection in free-ranging wild ruminants in Switzerland

**DOI:** 10.1186/1746-6148-9-166

**Published:** 2013-08-14

**Authors:** Julien Casaubon, Valérie Chaignat, Hans-Rudolf Vogt, Adam O Michel, Barbara Thür, Marie-Pierre Ryser-Degiorgis

**Affiliations:** 1 Centre for Fish and Wildlife Health (FIWI), Vetsuisse Faculty, University of Bern, Bern, Switzerland; 2 Institute of Virology and Immunoprophylaxis (IVI), Mittelhäusern, Switzerland; 3 Institute of Veterinary Virology (IVV), Vetsuisse Faculty, University of Bern, Bern, Switzerland

**Keywords:** Bluetongue virus, Cross-sectional study, Hemolysis, Switzerland, Toggenburg orbivirus, Wildlife samples

## Abstract

**Background:**

In 2006, bluetongue virus serotype 8 (BTV-8) was detected for the first time in central Europe. Measures to control the infection in livestock were implemented in Switzerland but the question was raised whether free-ranging wildlife could be a maintenance host for BTV-8. Furthermore Toggenburg orbivirus (TOV), considered as a potential 25^th^ BTV serotype, was detected in 2007 in domestic goats in Switzerland and wild ruminants were considered a potential source of infection. To assess prevalences of BTV-8 and TOV infections in wildlife, we conducted a serological and virological survey in red deer, roe deer, Alpine chamois and Alpine ibex between 2009 and 2011. Because samples originating from wildlife carcasses are often of poor quality, we also documented the influence of hemolysis on test results, and evaluated the usefulness of confirmatory tests.

**Results:**

Ten out of 1,898 animals (0.5%, 95% confidence interval 0.3-1.0%) had detectable antibodies against BTV-8 and BTV-8 RNA was found in two chamois and one roe deer (0.3%, 0.1-0.8%). Seroprevalence was highest among red deer, and the majority of positive wild animals were sampled close to areas where outbreaks had been reported in livestock. Most samples were hemolytic and the range of the optical density percentage values obtained in the screening test increased with increasing hemolysis. Confirmatory tests significantly increased specificity of the testing procedure and proved to be applicable even on poor quality samples. Nearly all samples confirmed as positive had an optical density percentage value greater than 50% in the ELISA screening.

**Conclusions:**

Prevalence of BTV-8 infection was low, and none of the tested animals were positive for TOV. Currently, wild ruminants are apparently not a reservoir for these viruses in Switzerland. However, we report for the first time BTV-8 RNA in Alpine chamois. This animal was found at high altitude and far from a domestic outbreak, which suggests that the virus could spread into/through the Alps. Regarding testing procedures, hemolysis did not significantly affect test results but confirmatory tests proved to be necessary to obtain reliable prevalence estimates. The cut-off value recommended by the manufacturer for the screening test was applicable for wildlife samples.

## Background

Bluetongue (BT) is a disease of economic importance [1] caused by the bluetongue virus (BTV), a RNA-virus that belongs to the genus *Orbivirus* of the family *Reoviridae*. Twenty-six serotypes have been reported around the world so far [2]. Although other infection pathways have been described [3,4], BTV is generally transmitted by biting midges (*Culicoides* spp.) [5]. The virus may cause a hemorrhagic disease with high morbidity rates, especially in sheep, while cattle mostly act as a reservoir. As an exception, a high morbidity was observed in this species during the recent epidemic due to BTV serotype 8 (BTV-8) in Europe [6]. Observations during previous BT outbreaks and experimental infections have shown that indigenous wild ruminant species may become infected with and without clinical manifestations and may therefore act as a virus reservoir [7-12].

Indigenous Swiss cattle and sheep breeds are highly susceptible to BTV infection and develop clinical signs [13,14]. The first BTV-8 infection in a domestic animal in Switzerland was diagnosed at the end of October 2007 [13] and in 2008, like in most European countries confronted to the BT epidemic, a large scale compulsory vaccination campaign was initiated to limit the expansion of the virus [15]. From 2007 to 2010, 76 local outbreaks have been reported in Swiss livestock [16]. A study in wildlife prior to the 2007 epidemic reported no evidence of BTV infection in Swiss red deer [17]. However, considering recent data from other European countries [8,18-20], an update of the situation including more species from the whole country was necessary to evaluate the potential role of wild ruminants in the BT epidemiology in Switzerland and assess whether they may represent a threat to the success of the control program in livestock. Furthermore, a new orbivirus named Toggenburg orbivirus (TOV) was detected in 2007 in healthy goats in Switzerland [21]. Since then, it has been shown that the TOV circulates in small domestic ruminants, especially in the southern Swiss canton of Ticino (TI), and the question was raised as to whether wildlife may be a reservoir for this virus [22,23]. A study addressing risk factors for TOV infection revealed a possible association with alpine pastures [24].

In this study, we estimated the prevalence of infections with BTV and TOV in roe deer (*Capreolus c. capreolus*), red deer (*Cervus e. elaphus*), Alpine chamois (*Rupicapra r. rupicara*) and Alpine ibex (*Capra i. ibex*) over two years (2009 to 2011). We considered potential risk factors for infection such as geographical location, altitude, animal species, age and sex, and we compared our results with data on domestic ruminants. Given that blood samples from hunted wildlife are often of poor quality (e.g. hemolysis), which may affect reactions in serological tests [25], we investigated the influence of hemolysis on our testing protocol. Our data show that wild ruminants are currently not a reservoir for BTV and TOV in Switzerland. We also confirm that recording serum quality of samples from hunted wildlife is essential to correctly interpret test results, and that the use of confirmatory tests is crucial to identify false positive results.

## Results

### BTV infections

**Figure 1:**
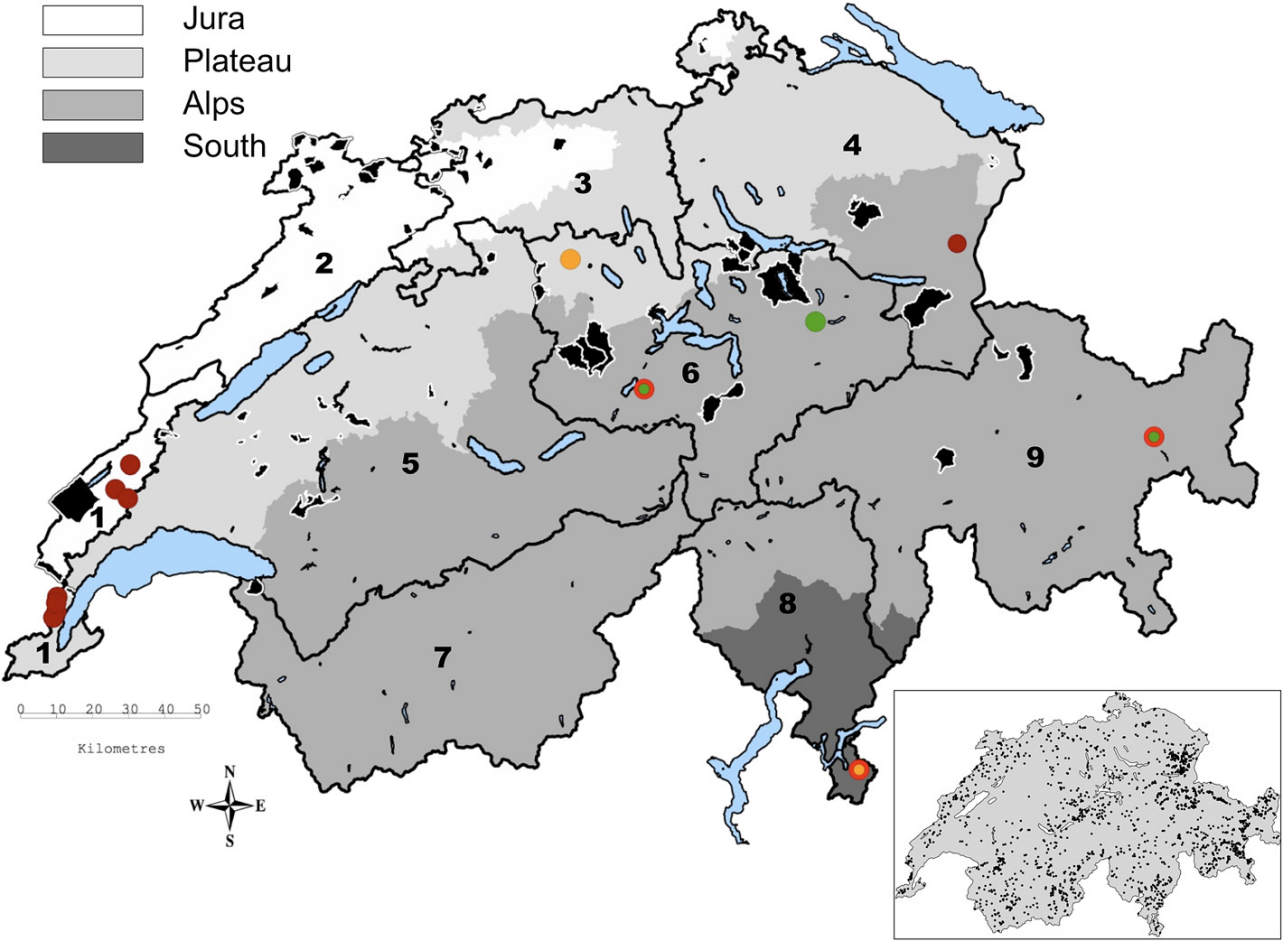
**Map of Switzerland showing the sampling regions and the distribution of the BTV-8 positive results.** Grey shaded areas represent different bioregions, major lakes are in blue. Numbers refer to sampling units: **1)** Jura-South, **2)** Jura-North, **3)** North-West, **4)** North-East, **5)** Centre-West, **6)** Centre-East, **7)** South-West, **8)** South-Centre, **9)** South-East. Colored dots without halo indicate the location of seropositive animals: Orange: roe deer; Dark red: red deer; Green: chamois; Yellow: ibex. Colored dots with a bright red halo correspond to PCR positive animals. Black areas are communities in which reported domestic BTV outbreaks occurred (data obtained from the Swiss Federal Veterinary Office). The framed small map of Switzerland in the right lower corner of the figure shows the sample distribution (black dots).

Out of 1,898 serum samples (439 roe deer, 480 red deer, 473 chamois, 506 ibex) screened for BTV antibodies with the VMRD ELISA, 118 showed an optical density percentage (ODP, inversely proportional to the optical density OD) value greater than 30%, which was the cut-off determined for this study. Ten of the 118 samples with an ODP > 30% (eight red deer, one roe deer and one chamois; Figure 1) were eventually confirmed seropositive by three additional ELISAs and by Serum Neutralization Test (SNT), with titers against BTV-8 ranging from 1:6 to 1:108 (Table 1 and 2). Only these confirmed positive samples were used for seroprevalence calculation. Overall, estimated seroprevalence was 0.5% (95% confidence interval [CI] 0.3-1%) (Table 1).

**Table 1:** Prevalences of antibodies against BTV-8 in four species of wild ruminants from Switzerland, 2009-2011

		**Sampling units**									
		**Jura**	**Jura**	**North**	**North**	**Centre**	**Centre**	**South**	**South**	**South**	**Total**
		**South**	**North**	**West**	**East**	**West**	**East**	**West**	**Centre**	**East**	
Roe deer	Seroprevalence	0	0	0	0	0	1.4	0	0	0	0.2
	(95% CI)	(0–13.7)	(0–5.3)	(0–6)	(0–4.5)	(0–9.5)	(0–7.5)	(0–8)	(0–20.6)	(0–9.7)	(0.01-1.3)
	positive / tested	0 /25	0 / 68	0 / 60	0 / 81	0 /37	1 / 72	0 / 44	0 / 16	0 / 36	1 / 439
Red deer	Seroprevalence	29.2	-	-	1	0	0	0	0	0	1.7
	(95% CI)	(12.6-51.1)			(0–5.3)	(0–7.4)	(0–8.4)	(0–6.5)	(0–4.5)	(0–2.8)	(0.7-3.3)
	positive / tested	7 / 24	-	-	1 /103	0 / 48	0 / 42	0 / 55	0 / 80	0 / 128	8 / 480
Chamois	Seroprevalence	0	0	0	0	0	1.5	0	0	0	0.2
	(95% CI)	(0–13.2)	(0–5.8)	(0–36.9)	(0–4.9)	(0–12.8)	(0.04-8.3)	(0–6.5)	(0–9.5)	(0–3)	(0.01-1.2)
	positive / tested	0 / 26	0 / 62	0 / 8	0 / 73	0 / 27	1 / 65	0 / 55	0 / 37	0 / 120	1 / 473
Ibex	Seroprevalence	-	-	-	0	0	0	0	0	0	0
	(95% CI)				(0–6.1)	(0–4.5)	(0–7.4)	(0–3.9)	(0–33.6)	(0–1.7)	(0–0.7)
	positive / tested	-	-	-	0 / 59	0 / 81	0 / 48	0 / 92	0 / 9	0 / 217	0 / 506
Total	Seroprevalence	9.3	0	0	0.3	0	0.9	0	0	0	**0.5**
	(95% CI)	(3.8-18.3)	(0–2.8)	(0–5.3)	(0.01-1.8)	(0–1.9)	(0.1-3.1)	(0–1.5)	(0–2.6)	(0–0.7)	(0.3-1)
	positive / tested	7 / 75	0 / 130	0 / 68	1 / 316	0 / 193	2 / 227	0 / 246	0 / 142	0 / 501	10 / 1,898

Samples are classified according to the nine sampling units. Confidence intervals (95% CI) are indicated in parentheses.

**Table 2:** Relationship between results of the serological tests and sample hemolysis

	**Score 0**		**Score 1**		**Score 2**		**Score 3**		**Total**	
	**n = 99**		**n = 338**		**n = 829**		**n = 576**		**n = 1842**	
	ODP	ODP	ODP	ODP	ODP	ODP	ODP	ODP	ODP	ODP
	30-49%	50-100%	30-49%	50-100%	30-49%	50-100%	30-49%	50-100%	30-49%	50-100%
**Screening ELISA**										
VMRD^a^	6	3	18	2	40	13	22	14	86	32
**Confirmatory ELISAs**										
VMRD^b^	0	3	0	0	0	3	1^c^	6	1^c^	12
BDSL	0	2	0	0	0	3	1^c^	4	1^c^	9
INGENASA	0	2	0	0	0	3	1^c^	3	1^c^	8
**Serum Neutralization Test**										
Titer ≥ 1:2	0	2	0	0	0	3	1	4	1	9
**Total seropositive**	**2**		**0**		**3**		**5**		**10**	
**Confirmed positive / screening positive (ODP ≥ 30%)**	**22.2%**		**0%**		**5.7%**		**13.9%**		**8.5%**	
**Seroprevalence**	**2.0%**		**0%**		**0.4%**		**0.9%**		**0.5%**	

^a^ Cut-off at ODP 30%, ^b^ Cut-off at ODP 50%, ^c^ Unclear.

Number of positive samples obtained with the screening test (VMRD ELISA) and following confirmatory tests are indicated in relation to the hemolysis serum scores (0–3) and the optical density percentage (ODP) of the screening test. One sample with ODP > 30% yielded an unclear result in the confirmatory ELISAs but to a positive result in the SNT (cut-off ≥ 1:2).

With the exception of one roe deer (fawn), all seropositive animals were adults (no statistically significant difference between age classes or sexes). There were significantly more seropositive red deer than roe deer (*p* = 0.039), chamois (*p* = 0.038) or ibex (*p* = 0.003). Among the sampling units, a significantly higher seroprevalence was recorded in Jura-South (9.3%, Table 1; *p* < 0.001 to *p* = 0.014), and within this unit, more red deer than roe deer and chamois (*p* = 0.004) were seropositive. The elevation above sea level (a.s.l.) of the seropositive animals did not differ from the species-specific elevation range for each ruminant species (Table 3). There was also no difference between the two sampling periods (2009 and 2010).

**Table 3:** Sample size and population data for the four wild ruminant species sampled in the study

**Species**	Geographical data*				Population data 2010**	
	**Bioregion**	**Min**	**Mean altitude m.a.s.l. (SD)**	**Max**	**Estimated population size**	**Hunting bag**
Roe deer	Jura	393	783 (231)	1,228	112,975	39,664
	Plateau	355	553 (126)	938		
	Alps	374	1,195 (445)	2,504		
	South	272	731 (337)	1,417		
Red deer	Jura	474	764 (375)	1,557	28,483	9,016
	Alps	402	1,254 (399)	2,604		
	South	238	940 (465)	2,094		
Alpine chamois	Jura	434	940 (279)	1,581	91,390	13,339
	Plateau	532	650 (160)	1,007		
	Alps	431	1,840 (547)	3,103		
	South	312	1,164 (540)	2,087		
Alpine ibex	Alps	673	2,294 (392)	3,154	15,553	1,074

* Data from the animals sampled in this study.

** Data obtained from the Swiss Federal Office of the Environment [55].

Population size is based on counts and estimates from wildlife managers and biologists.

Mean altitudes differed significantly between species (p < 0.05): Ibex were found higher than all other species (Alps), and chamois were higher than roe deer and red deer (all bioregions).

None of the 10 seropositive samples was positive by S10 BTV real-time RT-PCR. In contrast, BTV-8 RNA was detected in 3 out of 1,070 seronegative samples (Ct-values between 28 and 31). These three samples were from two adult chamois out of the 118 samples with initial positive reaction in the VMRD ELISA screening (ODP > 30%) and from one adult roe deer from the canton of Ticino (sampling unit South Centre, Figure 1) that tested negative in the VMRD ELISA screening. Virus isolation from these three samples was not successful. Overall, BTV-8 virus prevalence was 0.3%, (95% CI 0.1-0.8%) and no animal was found positive for TOV.

The comparison of our results with documented BT outbreaks in domestic livestock (virus positive animals, data from the Swiss Federal Veterinary Office; Figure 1) shows that seropositive wild ruminants were found in a range from 2.0 to 7.3 km (average of 5.8 km, linear distance) around communities with domestic outbreaks. In contrast, two virus positive animals (one chamois and one roe deer) originated from regions where no domestic BT outbreak has been reported so far [16], at a distance of 38.0 km and 85.5 km, respectively, to the next community with a documented outbreak.

### Serum quality and testing protocol

**Figure 2:**
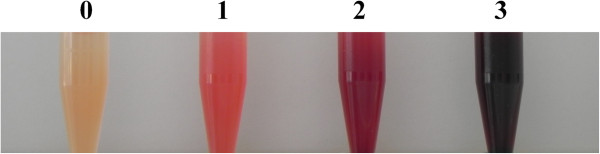
**Typical appearance of thawed serum samples and attributed hemolysis scores.** 0 = clean and transparent; 1 = mildly hemolytic, transparent; 2 = moderately hemolytic, decreased transparency; 3 = severely hemolytic and opaque.

The time span between sample collection in the field and arrival at laboratory was in average 3.2 days with a range of 0 to 21 days. Of all obtained serum samples, 1,842 were scored (0 to 3) according to their degree of hemolysis (Figure 2). Only 5% were scored as clean (0) and 18% as mildly hemolytic (1). Most of the samples (45%) were classified as moderately hemolytic with decreased transparency (2), while 31% were severely hemolytic and opaque (3). As expected, the proportion of clean samples was higher in animals sampled alive (55%) than after death (4%; *p* < 0.001), while the proportion of moderately to severely hemolytic samples was similar in hunted animals (77%) and animals found dead (70%). The distribution of the different hemolysis scores was generally similar in all four species. However, the proportion of clean samples was significantly higher in hunted ibex (7%) than hunted chamois (2%, *p* = 0.001), despite the fact that the time span (in days) between the date of sampling and the date of arrival at the Centre for Fish and Wildlife Health (FIWI) was longer for samples of hunted ibex (3.3 days, range of 1 to 19 days) than of hunted chamois (3.1 days, range of 0 to 21 days) (*p* = 0.012). Nevertheless, when the animal species was not considered, hemolysis generally increased with the time between sampling and sample processing (2.8 days for score 0, and 3.6 for score 3).

**Figure 3:**
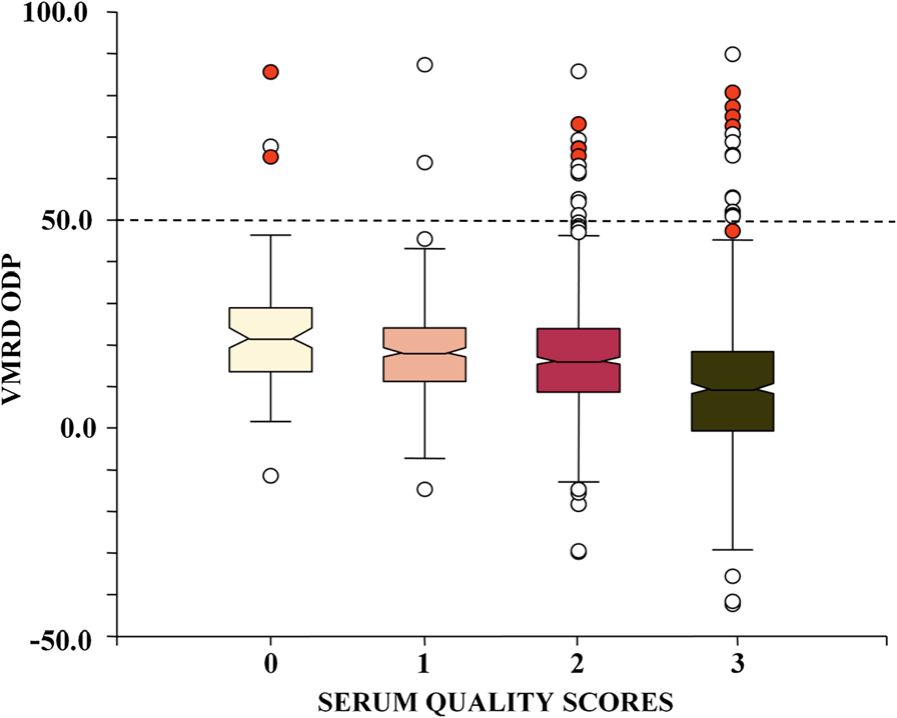
**Box plots of the VMRD ELISA results in relation to serum quality.** Red dots represent the BTV-8 seropositive animals as confirmed by SNT. Dashed line indicates the 50% cut-off value defining a positive sample as recommended by the manufacturer.

In the screening VMRD ELISA, severely hemolytic samples (score 3) showed a significantly lower ODP mean value than samples with lower scores (*p* < 0.001; Figure 3), and the overall range of ODP values was wider with increasing hemolysis. Positive results were confirmed both for clean and hemolytic sera (Figure 3). Final seroprevalence was 2.0% for clean samples (score 0) and 0.6% for markedly hemolytic samples (score 2 and 3), without statistical difference between the two groups, and with even a lower prevalence for only slightly hemolytic samples (0%, score 1; Table 2).

Ten out of 118 samples (8.5%) were eventually confirmed seropositive by repeating the VMRD ELISA in a different laboratory by other technicians and by two other ELISAs and SNT (Table 2). The percentage of confirmed positive among screening-positive samples was higher for score 0 (22.2%) than for scores 2 and 3 (5.7% and 13.9%, respectively) but these differences were not significant. Overall, final seroprevalence (10/1,898, 0.5%) was significantly lower than the initial one (screening test: 118/1,898, 6.2%; *p* < 0.001) even if considering only samples with ODP > 50% (9/1,898 confirmed versus 32/1,898 positive in the screening test, *p* < 0.001).

Except for one out of the 10 samples confirmed as positive after running confirmatory tests, all of these had an initial ODP > 50% (Table 2, Figure 3). Thus, the estimated seroprevalence using 50% as cut-off (9/1,898, 0.47%) would have not significantly differed from the one obtained with the cut-off at 30% (10/1,898, 0.52%; *p* = 1).

## Discussion

Following the recent BT epidemic in central Europe, data on BTV circulation in Swiss free-ranging wild ruminants were urgently needed for the future planning and successful achievement of the implemented BT control program in livestock. This study provides an overview of the situation in the four indigenous wild ruminant species in Switzerland, and it additionally addresses important questions regarding testing protocols for samples collected from wildlife carcasses.

The low estimated seroprevalence for BTV in all investigated species suggests that sporadic infection can occur but that BTV-8 is not circulating within wild populations in Switzerland at a large scale. Also, the prevalence in red deer of 1.7% in the present study was not significantly higher (*p* = 0.058) than reported before the European epidemic (0%, CI 0–1.54%) [17].

The fact that the prevalences in our study were lower than the ones previously documented in other European countries [8,19,20] can be due to two different factors. First, albeit 76 BT outbreaks have been recorded in Swiss livestock between 2007 and 2010 [16], this number is very low compared to the situation observed in other countries [26], e.g. 26,500 herds with clinical cases in 2008 in France [27]. The virus has apparently not spread as widely in Switzerland as in other European regions. Second, our sampling rounds took place after two years of mandatory vaccination of Swiss domestic ruminant species [15], a measure that limited the incidence of BTV-8 infections in domestic and possibly also in wild species [8,28].

In our study, red deer were more often infected by BTV-8 than other wild ruminant species, similarly to observations in Belgium [8], Spain [19], and France [20]. This supports the hypothesis that, although they live in the same habitat, red deer are more susceptible to infection than roe deer. It is interesting to note that red deer are abundant in the southern part of the Swiss Jura while only few individuals are present in its northern part [29], where most of the domestic Swiss BT outbreaks occurred [16]. In contrast, roe deer and chamois are abundant in the northern Jura [29] but none of the individuals from this region (133 samples) were positive. This further suggests that ruminants other than red deer have a lower susceptibility to infection or that BTV-infected midges do not feed on them as often. In conclusion, it indicates that outbreaks in livestock were likely not related to wildlife infections.

It has been formerly of concern that BTV may expand from southern Europe through the Alps but data from 2004–2005 did not indicate virus presence in regions considered at risk in the Swiss Alps [17]. Interestingly, in our study we found a chamois positive by real-time RT-PCR, originating from an Alpine valley (Engadin, canton of Graubünden), which was sampled above 2,000 m a.s.l. To our knowledge, this is the first report of BTV-8 RNA in an Alpine caprid. It has been shown that there are no vector-free areas in Switzerland, and different species of *Culicoides* midges have been detected up to 2,100 m a.s.l. in the Swiss Alps, indicating that virus may be able to circulate within the Alps [30]. Although anecdotal, our result supports this hypothesis.

During previous BT epidemics in Europe, the situation in wildlife seemed to be closely related to the spread of BTV among domestic livestock [18]. However, seropositive wild animals have also been documented in regions without domestic outbreaks [31], a situation that was particularly obvious for BTV-8 in southern Spain [19]. In contrast to Spain, where wild ruminants may act as reservoir for BTV [19,31], our data indicate that wildlife plays currently no such role in Switzerland. First, seroprevalences in wildlife are very low; second, all seropositive wild ruminants were sampled in a perimeter of < 5.8 km around a domestic outbreak, while 95% of the new domestic outbreaks during the 2006 BT epidemic occurred within a perimeter of 31 km from the first one [32]. In our study, two infected wild ruminants were found in areas distant of more than 30 km from the nearest Swiss domestic case (38.0 km and 85.5 km, respectively) but virus dispersion over large distances through wind transport of infected midges is well known [31]. Alternatively, infected domestic animals in these areas may have remained undetected or unreported. The detection of clinically ill animals in rigorously vaccinated herds and the health monitoring of livestock kept on alpine pastures are challenging. Furthermore, one of the two positive wild ruminants originated from a location close to the Italian border and may have been related to domestic cases in northern Italy. Indeed, BTV-8 cases have been recorded in Piemonte [33] but to our knowledge, detailed information on the epidemiological situation in this region is not publicly available. Nevertheless, these two virus positive animals demonstrate that wild ruminants, as it has already been addressed [34], should imperatively be included in future BT surveillance programs for early detection of disease.

Concerning TOV, our study suggests that wildlife is not a reservoir for this virus either. Despite the high apparent TOV seroprevalence in goats (49% at individual level and of 60% at herd level) in the canton of Ticino and the fact that TOV had circulated in the population of domestic small ruminants in 2008 and 2009 [22], TOV-positive wild animals were not found in this region.

Serological surveys in free-ranging wild species are linked with a range of difficulties including laborious sampling (few samples obtained despite intensive capture efforts, dependence of hunters and gamekeepers for access to hunted animals), limited representativity of obtained sample sets (sampling bias due e.g. to hunting or capture season and hunting plans), poor sample quality (hemolysis, contamination of different origin), and lack of validation of serological tests for wild species. Poor serum quality can hamper analysis and complicate result interpretation. However, the impact of sample quality depends largely on the type of serological test. Hemolysis has been shown to be a major problem when using serum neutralization tests (SNT) due to serum cytotoxicity and to the presence of cloudy suspensions interfering with the reading of the test [19]. Hemolytic and strongly contaminated samples are also considered inadequate for the complement fixation test and serum agglutination test [35], and decreased sensitivity due to hemolysis was observed when testing samples of wildcats with an immunofluorescence assay [36] and fallow deer sera with an ELISA [37]. Similarly, a recent study on Suid Herpesvirus 1 showed that hemolysis and repeated freeze-thawing cycles of wild boar sera can influence ELISA test reactions, leading to a higher number of doubtful results [24]. In contrast, hemolysis and repeated freeze-thawing cycles have been reported to have only a relatively small effect on the obtained optical density (OD) values of an ELISA applied on domestic pig sera [38]. However, it is difficult to evaluate which factor is most important when the impact of hemolysis on test results is addressed in association with suboptimal storage conditions. For example, long sample storage time at room temperature has been shown to increase the number of false negative results [39]. Here, we evaluated the influence of hemolysis on serological tests in four different wild ruminant species. As expected, the majority of the samples collected from hunted and perished animals was moderately to severely hemolytic, in contrast to samples from living animals. We documented that the range of ODP values was wider and the mean ODP value lower in case of severe hemolysis, indicating a higher risk of obtaining false negative results when applying recommended cut-offs on hemolytic samples. However, only one hemolytic sample with ODP < 50% was confirmed positive in other tests, suggesting that false negative results due to hemolysis is negligible. Nevertheless, this result should be considered with caution given the very low number of positive samples in our study.

Our testing protocol, which combined an initial highly sensitive screening procedure with more specific confirmatory tests, was intended to detect weak positive antibody reactions as expected after a recent BTV infection or in TOV-infected animals. This procedure allowed us to identify such samples to test them individually for virus RNA, while seronegative samples were tested in pools. Independently of the degree of hemolysis, a high number of samples identified as positive in the screening test turned out to be negative in the confirmation tests, which was due in large part to the low cut-off value set in the screening test. However, even when considering only samples with an ODP > 50%, the use of confirmatory tests proved to be essential to minimize the number of false positive reactions. Based on our experience, we recommend re-testing samples showing a weak positive or questionable result. If samples are truly only weakly positive, they will produce the same reaction in the second run; in contrast, false positive results are mostly not repeatable. Despite the known limitations of the SNT when applied on hemolytic samples, we obtained acceptable results in all cases. This success is probably due to our use of a lower TCID_50_ than the one recommended by the OIE, namely 20–40 TCID_50_ instead of 100 TCID_50._ This protocol modification has been shown to be more sensitive without a loss of specificity for the detection of antibodies against BTV [40]. The serial serology testing procedure privileged specificity because only samples that scored positive in at least two out of three confirmatory ELISAs as well as in the SNT were considered positive. However, sample selection for investigation by real-time RT-PCR was based on the results of the screening test only, increasing the chances of detecting viremic animals.

Three samples determined as seronegative were positive for BTV RNA by real-time RT-PCR. In the case of BTV infection, viremia can be detected up to a couple of months after infection in domestic and wild species, usually in seropositive animals [12]. However, the virus may be occasionally found in seronegative individuals, as it has been reported in red deer from Belgium [8]. This could be either related to a recent infection (before seroconversion) or due to a failure in antibody detection (false seronegative).

Pooling seronegative samples for PCR analyses may have decreased sensitivity of the test. However, we were looking for epidemiologically relevant virus amounts associated with a clearly positive reaction; even in the case of a low virus load, we would have expected the animals to be seropositive. Therefore, the pooling procedure allowed saving resources without affecting result quality. While the storage of blood samples at −20°C is not considered problematic for the real-time RT-PCR, virus isolation could have been negatively influenced by this temperature. This may explain the failure of isolation from the three positive RT-PCR samples.

## Conclusions

Our aim was to assess the role of wild ruminants in the epidemiology of BTV infections in domestic livestock in Switzerland. Our data suggest that BTV infections in wild ruminants are only sporadic and that this virus does not circulate among wild populations. We therefore conclude that wildlife is currently an incidental spill-over and not a maintenance host in Switzerland and does not represent a threat for the BTV control program in livestock. Similarly, we found no evidence of TOV infections in wild hosts. However, the presence of BTV-8 in areas distant from reported cases in domestic species suggests that the BTV-8 situation may evolve in the near future and that wild ruminants could be used as sentinels for BT surveillance. Additionally, our data suggest that hemolytic samples can be used for serosurveys, but they underline the importance of running confirmatory tests to refine results obtained by sensitive screening procedures.

## Methods

### Study design and sampling strategy

This study was based on a cross-sectional convenient sampling strategy aiming also at estimating the apparent prevalence of infections with the bovine viral diarrhea virus in Swiss wild ruminants [41]. The whole territory of Switzerland (41,285 km^2^) was divided in nine sampling units (Figure 1) based on definitions for the BT monitoring program in Swiss livestock [42], political units (cantons), environmental factors influencing the probability of vector occurrence [43], the occurrence of wild ruminants, and documented BTV and TOV infections in livestock. Additionally, the altitude range (AR) of the main four Swiss bioregions was determined to assess the potential role of altitude as a risk factor for infection: Jura: 273–1,679 m a.s.l., Plateau: 244–1,290 m a.s.l., Alps: 263–4,634 m a.s.l., South: 193–2,887 m a.s.l. We also calculated the mean altitude of sampling locations for each species in each bioregion (Table 3).

Sampling was carried out from August 2009 to April 2011, and the required sample size for “detection of disease” was calculated with the WinEpiscope 2.0 software package [44], assuming an expected maximal prevalence of 1% for virus positive animals, with a confidence interval of 95% and an accepted error of 5%. We aimed at a total of 300 animals per species and year [41].

### Sample collection and animals

Blood samples were collected between 2009 and 2011. This study did not involve purposeful killing of animals and was exempt from ethical approval according to Swiss legislation. Samples originated mainly from dead wildlife legally hunted during the hunting season (922.0 hunting law; for details on sampling organization, see [41]). Few animals submitted for necropsy as carcasses to the Centre for Fish and Wildlife Health (FIWI) or captured in the fields in the frame of other wildlife projects, were also sampled and included in the study. Capture and sampling of living animals (n = 24) were carried out with the authorizations of the competent authorities, as requested by Swiss legislation (455 animal protection law, including legislation on animal experimentation; authorization numbers GR 24/06 and VD 1863). Sampling conditions (hunt, found dead, capture), sampling date, and biological data were recorded for each animal. In dead animals, blood was collected from the heart or from body cavities. In living animals, blood was drawn from the jugular vein under anesthesia. Blood samples were transferred into tubes with and without anticoagulant (EDTA), sent to the laboratory, and serum samples were centrifuged immediately upon reception. Aliquots of sera and whole blood were stored at −20°C until analysis.

Blood samples of 1,898 wild ruminants were collected from all over the country. Slightly more males (n = 1,012) than females (n = 878) were sampled, and information on sex was missing for eight animals. The majority of the samples came from adult individuals (≥ 2 years; n = 1,365), followed by yearlings (1 to < 2 years; n = 302) and fawns/kids (< 1 year; n = 221). Information on age was missing for 10 animals. Depending on available blood quantity, samples were tested for BTV antibodies and/or for the presence of viral RNA.

### Laboratory analyses

Prior to serological screening tests, thawed serum samples were scored according to their color and opacity. (0 = clean and transparent; 1 = mildly hemolytic, transparent; 2 = moderately hemolytic, decreased transparency; 3 = severely hemolytic and opaque; Figure 2).

For the detection of BTV-specific antibodies, serum samples were first screened with a commercial competitive ELISA kit (Bluetongue Virus Antibody Test Kit, cELISA, VMRD, Inc., Pullman, U.S.A.; referred to as VMRD ELISA) that detects antibodies against all BTV-serotypes [45] including BTV-26 and TOV [22]. The test was performed according to the manufacturer’s instructions but we used the ODP instead of the OD to classify results. A sample is positive if it produces an OD lower than 50% of the mean of the negative controls; when using ODP values, a sample is positive when the ODP value is equal to or higher than 50% of the mean of the negative controls. However, for the screening test, we decided to set the cut-off at an ODP value of 30% because we wanted to detect recently infected animals displaying a weak antibody reaction below the normal cut-off and additionally test them for BTV by real-time RT-PCR. Reactions to anti-TOV antibodies are often weak and early time-point seroconversions are rarely detected with normal testing procedures [23,46]. Furthermore, this test has not yet been validated for wild species and it is not known whether the antibody reaction with samples from wildlife is as strong as with samples from domestic animals. For seroprevalence estimation, positive samples were subsequently re-analyzed with the VMRD ELISA following manufacturer’s recommendations and with two other kits: another competitive ELISA (Blue Tongue Competitive ELISA Kit, B.D.S.L, Irvin, Scotland, UK; referred to as BDSL ELISA) and an ELISA based on the double recognition principle (INGEZIM BTV DR, INGESANA, Madrid, Spain; referred to as INGENASA ELISA). Samples that yielded positive results in at least two different confirmatory ELISAs were subsequently analyzed with a SNT specific for BTV-8 antibodies. Briefly, two-fold serial dilutions of serum samples starting at 1:2 were made in 96-well plates. Each dilution was titrated against the Northern European BTV-8 strain (kindly provided by the Friedrich-Loeffler-Institute, Riems, Germany) by adding an equal volume of virus solution containing 20–40 TCID_50_/50 μL. The 96-well plates were incubated for 1 h at 37°C and 5% CO_2_. Then, 100 μL of a 1.5 × 10^5^/mL Vero cell suspension were added per well, and after incubation for 4 to 7 days at 37°C and 5% CO_2_, the wells were scored for cytopathic effect. The neutralization titer was determined as the dilution of serum yielding a 50% neutralization end point. Any neutralization was considered to be positive.

Positive samples in the first VMRD ELISA (cut-off set at 30%) were tested individually by real-time RT-PCR. Additionally a subset of seronegative samples was tested in pools of five samples each, including all available samples collected in 2009 from all sampling units as well as those collected in 2010 from the sampling unit South (canton of Ticino), because numerous TOV-infected goats were found in this region [24]. Total RNA was purified following a combination of a manual protocol using TRIzol® (Life Technologies Ltd, Paisley, UK) and a semi-automatic commercial extraction kit (NucleoSpin® 96 RNA, Marcherey-Nagel, Düren, Germany) as previously described [47]. Briefly: 250 μL EDTA-blood was mixed with 750 μL TRIzol® and 10 μL glycogen (Fluka-Chemie AG, Buchs, Switzerland) and an internal positive control to detect PCR inhibition. After adding 200 μL chloroform (Merck, Darmstadt, Germany) and centrifugation step according to the published protocol [47], the upper phase was mixed 1:1 with ethanol 100%. In a second step, this mixture was used as matrix for an automated RNA extraction using NucleoSpin® 96 RNA kit. The RNA was then tested by real-time RT-PCR employing the S10-specific protocol able to detect all BTV-serotypes, inclusively TOV [48]. In S10-specific real-time RT-PCR positive samples, a serotype-8 specific commercial real-time RT-PCR was performed (TaqVet® Blue Tongue Virus Triplex – All Genotypes and BTV8, LSI, Lissieu, France) and if negative a TOV specific real-time RT-PCR was carried out [46]. In the case where a pool yielded a positive result, samples within that pool were additionally tested as single reactions. For virus isolation, washed blood or cell culture supernatant were inoculated intravenously into 10–12 days old SPF embryonated chicken eggs as described by the OIE Guidelines [49].

### Data management and statistical analysis

Data handling, validation, cleaning and coding were done in MS Excel© spread sheets followed by transfer to the NCSS 2007 software (Hintze, J. (2007). NCSS 2007. NCSS, LLC. Kaysville, Utah, USA. http://www.ncss.com) for statistical analyses. Prevalences were calculated assuming test sensitivity and specificity of 100%. The two-tailed Fischer’s exact test (FET) was used to determine differences in prevalence of infection among age classes, sexes, geographical regions and sampling periods. The Mann–Whitney U test was used to test differences between means of altitude. Both tests were applied to study differences between means of optical density percentage (ODP) of the VMRD ELISA among sera with different scores and among species. Level of significance was set at *p* < 0.05. Non-interpretable serological and PCR results were not included in the statistical analyses.

Maps were designed using the gvSIG software, version 1.11.0 final (© gvSIG Association). Elevation of animal location at sampling time as well as altitude range of the four Swiss bioregions (based on the definition of the Federal Office of the Environment [50]) were calculated with the ArcView GIS software 3.0a and adapted on the basis of appropriate literature [51].

## Competing interest

The authors declare that they have no competing interest.

## Authors’ contributions

JC contributed to sample collection, performed the serological screening tests, analyzed the data, and drafted the manuscript. VC carried out the serological confirmation tests and supervised the real-time PCR. HRV contributed to the study design and supervised the serological screenings. AOM organized the sample collection and contributed to serological screening tests. BT contributed to the study design and supervised the confirmation tests. HRV, BT and VC contributed to the interpretation of laboratory results. MPRD designed and coordinated the study, contributed to sample collection and data analysis, and drafted the manuscript. All authors critically read and approved the final manuscript.
